# Parkinson’s Disease Diagnosis and Severity Assessment from Gait Signals via Bayesian-Optimized Deep Learning

**DOI:** 10.3390/diagnostics15162046

**Published:** 2025-08-14

**Authors:** Mehmet Meral, Ferdi Ozbilgin

**Affiliations:** 1 Department of Neurosurgery, Private Erciyes Hospital, Kayseri 38020, Türkiye; m.meral@erciyeshastanesi.com.tr; 2 Department of Electrical and Electronic Engineering, Giresun University, Giresun 28200, Türkiye

**Keywords:** Parkinson’s disease, gait analysis, deep learning, Bayesian optimization, diagnostic

## Abstract

**Background/Objectives**: Early diagnosis of Parkinson’s Disease (PD) is essential for initiating interventions that may slow its progression and enhance patient quality of life. Gait analysis provides a non-invasive means of capturing subtle motor disturbances, enabling the prediction of both disease presence and severity. This study evaluates and contrasts Bayesian-optimized convolutional neural network (CNN) and long short-term memory (LSTM) models applied directly to Vertical Ground Reaction Force (VGRF) signals for Parkinson’s disease detection and staging. **Methods**: VGRF recordings were segmented into fixed-length windows of 5, 10, 15, 20, and 25 s. Each segment was normalized and supplied as input to CNN and LSTM network. Hyperparameters for both architectures were optimized via Bayesian optimization using five-fold cross-validation. **Results**: The Bayesian-optimized LSTM achieved a peak binary classification accuracy of 99.42% with an AUC of 1.000 for PD versus control at the 10-s window, and 98.24% accuracy with an AUC of 0.999 for Hoehn–Yahr (HY) staging at the 5-s window. The CNN model reached up to 98.46% accuracy (AUC = 0.998) for binary classification and 96.62% accuracy (AUC = 0.998) for multi-class severity assessment. **Conclusions**: Bayesian-optimized CNN and LSTM models trained on VGRF data both achieved high accuracy in Parkinson’s disease detection and staging, with the LSTM exhibiting a slight edge in capturing temporal patterns while the CNN delivered comparable performance with reduced computational demands. These results underscore the promise of end-to-end deep learning for non-invasive, gait-based assessment in Parkinson’s disease.

## 1. Introduction

Parkinson’s disease (PD) is the second most common neurodegenerative disorder worldwide, affecting over 10 million individuals globally, with its prevalence expected to double by 2040 due to the aging population [1]. The disease is primarily associated with progressive degeneration of dopaminergic neurons in the substantia nigra, leading to a wide range of motor symptoms such as bradykinesia, resting tremors, muscular rigidity, and postural instability. These motor impairments significantly reduce the quality of life of patients and create substantial socioeconomic burdens on healthcare systems worldwide [2].

Despite significant advances in neurology and neuroimaging, the diagnosis of PD remains primarily clinical, based on observable motor symptoms and validated scales such as the Unified Parkinson’s Disease Rating Scale (UPDRS) and the Hoehn and Yahr (HY) staging system [3,4]. However, these scales are subjective, and early motor symptoms can be subtle and easily overlooked. This often leads to delayed diagnosis, by which time more than 50% of dopaminergic neurons may already be lost [5]. One of the earliest and most telling signs of PD is abnormal gait. Changes such as reduced stride length, increased stance time, irregular cadence, and variability in step symmetry are commonly observed in PD patients [6]. These gait anomalies can be objectively captured using modern sensor technologies, providing a non-invasive and data-driven alternative to traditional clinical assessments. In particular, Vertical Ground Reaction Force (VGRF) sensors have demonstrated exceptional promise in quantifying gait dynamics with high temporal resolution and sensitivity.

Timely and accurate diagnosis is critical in medical practice, as it allows for early intervention, which can significantly improve patient outcomes and reduce disease-related complications [7,8,9]. In many conditions such as PD, where the onset of symptoms is gradual and often subtle, detecting abnormalities at an early stage remains a major clinical challenge. By the time hallmark motor impairments become observable, a significant portion of neuronal loss has typically already occurred, limiting the effectiveness of available treatments. This has led to a growing emphasis on the development of objective, sensitive, and non-invasive tools for early detection in clinical and research settings. Various studies have explored this goal for PD, employing data from vocal characteristics, handwriting dynamics, and gait signals, all of which reflect motor impairments that may precede clinical diagnosis [10,11,12].

A diverse body of research has focused on leveraging machine learning (ML) and deep learning (DL) algorithms to extract discriminative features from such modalities and enhance the accuracy of PD detection. Jang and Lee [13] proposed a neuro-fuzzy system incorporating fast Fourier transform (FFT) and principal component analysis (PCA), achieving moderate classification performance. Wu and Krishnan [14] employed least squares support vector machines (LS-SVM) combined with stride interval variability, reaching over 90% accuracy but requiring substantial computational resources. Similarly, Lee and Lim [15] used wavelet-based features alongside neural networks and obtained satisfactory results, though their model was sensitive to noise and signal non-stationarity. In another approach, Daliri [16] applied a chi-square kernel with short-time Fourier transform features to classify gait data using SVMs, demonstrating strong accuracy with expert-driven tuning. Zeng et al. [17] integrated phase space reconstruction and empirical mode decomposition (EMD) with neural networks, successfully capturing nonlinear gait dynamics. More recent works have explored advanced statistical descriptors and local binary pattern (LBP) variants for feature extraction [18,19], though these handcrafted approaches often fall short in capturing the variability inherent in pathological gait.

Deep learning models, particularly convolutional neural networks (CNNs), recurrent neural networks (RNNs), and their hybrids, have become increasingly prominent due to their capacity to learn hierarchical and temporal features directly from raw gait signals. Yurdakul et al. [20] and Jane et al. [21] reported high classification accuracies exceeding 98% by combining CNNs with neighborhood representation features, albeit with high preprocessing demands. Similarly, El Maachi et al. [22] applied a 1D-CNN model to VGRF data to predict PD severity based on the UPDRS scale, while Zhao et al. [23] integrated CNN and long short-term memory (LSTM) networks to improve temporal sensitivity, reporting accuracies ranging from 93% to 100% depending on the validation strategy. Aşuroğlu and Oğul [24] further demonstrated that combining CNN with wavelet-based feature reconstruction significantly enhanced performance, achieving 99.1% classification accuracy with perfect sensitivity and specificity. These results highlight the promise of leveraging raw VGRF signals in deep learning frameworks, eliminating the need for intermediate decomposition. However, while optimized CNN and LSTM networks have achieved strong results in various time-series applications, their individually optimized performance on raw VGRF data has yet to be benchmarked—a gap that the present study fills by implementing and contrasting both architectures for PD detection and staging.

While several studies have leveraged decomposed gait signals frequently applying a single technique such as fast Fourier transform, wavelet transform, or empirical mode decomposition for feature extraction, this strategy may only partially reflect the rich temporal dynamics inherent in raw VGRF waveforms and often entails considerable preprocessing. Moreover, although deep learning architectures like CNNs and LSTMs have been employed in this domain, comprehensive, head-to-head comparisons of their Bayesian-optimized variants remain relatively uncommon. Finally, few investigations have sought to develop an end-to-end framework capable of both PD detection and severity staging directly from unprocessed VGRF data. The novelty of this work lies in combining end-to-end learning from VGRF signals with systematic Bayesian optimization for both CNN and LSTM architectures, each analyzed independently to assess their respective strengths. This design enables a direct, fully automated pipeline for simultaneous PD detection and severity staging without manual feature engineering, while minimizing preprocessing requirements. The present work aims to address these areas by applying Bayesian-optimized CNN and LSTM networks to raw VGRF time-series and systematically contrasting their performance in a unified evaluation.

In this work, we introduce an end-to-end deep learning framework that directly leverages raw VGRF time-series for both PD classification and severity staging. Rather than relying on manual feature engineering or signal decomposition, we segment each VGRF recording into fixed-length windows, apply standard normalization, and feed the resulting sequences into two separate model pipelines: a CNN and a LSTM network. Each architecture is fine-tuned via Bayesian optimization to identify optimal hyperparameter settings, ensuring robust learning of spatial–temporal and sequential patterns, respectively. We evaluate both models on publicly available VGRF datasets and present a systematic, head-to-head comparison of their performance in terms of accuracy, precision, recall, F1-score, and AUC. The results demonstrate that our Bayesian-optimized, raw-signal approach not only simplifies the preprocessing workflow but also achieves competitive or superior classification and staging accuracy compared to existing methods.

The remainder of this paper is structured as follows: Section 2 describes the dataset and the end-to-end methodology, detailing raw VGRF signal preprocessing (windowing and normalization), the design of standalone CNN and LSTM, and the Bayesian optimization procedure for hyperparameter tuning. Section 3 reports the experimental setup and comparative classification and staging results on benchmark VGRF datasets. Section 4 discusses these findings in the context of related work and highlights the strengths and limitations of each architecture. Finally, Section 5 concludes the paper and suggests avenues for future research.

## 2. Materials and Methods

**Figure 1:**
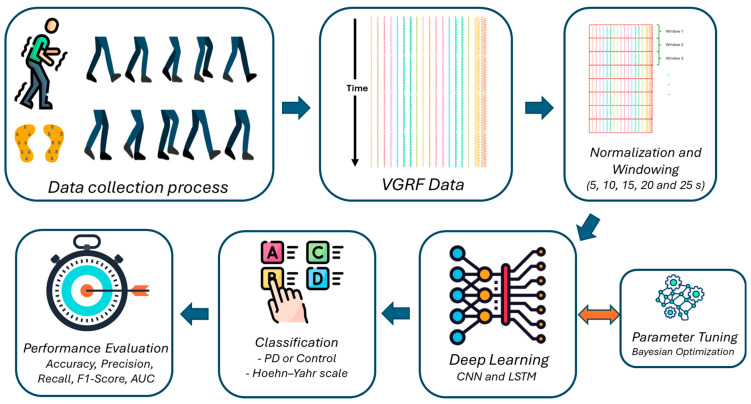
Proposed methodology.

This section comprises the materials and methods of the study, briefly introducing the gait dataset employed for PD classification, summarizing key preprocessing and outlining the deep learning framework used to analyze the processed VGRF signals. An overview of the complete workflow is depicted in Figure 1.

### 2.1. Dataset

**Figure 2:**
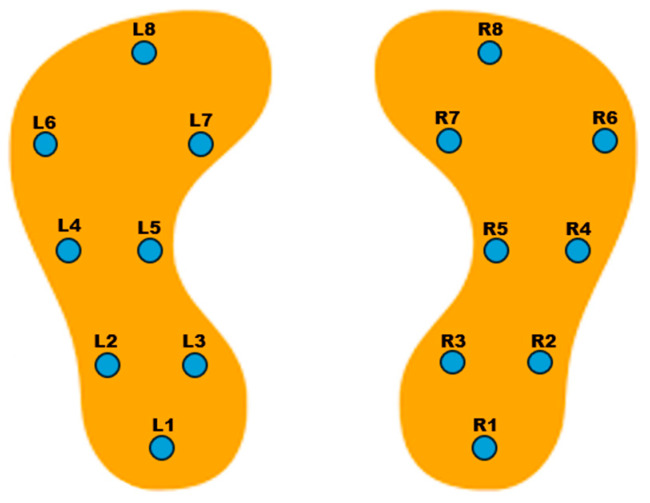
Sensor placements on the feet.

This study analyzes de-identified data from the publicly available “Gait in Parkinson’s Disease” (gaitpdb) database on PhysioNet. The original data collection was conducted under institutional review board oversight, and participants provided written informed consent, as reported in prior publications using this dataset. Our analysis involved only secondary use of de-identified public data; no new data were collected and no re-identification was attempted [25]. It includes VGRF signals recorded from a total of 166 individuals, comprising 93 patients diagnosed with PD and 73 healthy control (HC) subjects [11,26]. Data collection was performed using instrumented insoles equipped with eight force sensors under each foot. The sensors captured the VGRF values at a sampling rate of 100 Hz, providing precise temporal resolution of foot-ground contact during walking. The sensor arrangement followed a fixed coordinate scheme, enabling consistent interpretation of spatial force distributions across subjects [25,27]. As illustrated in Figure 2, the sensors were distributed systematically beneath both feet to capture biomechanical pressure patterns. Each data file consists of 19 columns: the first column corresponds to time (in seconds), columns 2 through 9 represent the force data from the left foot sensors, columns 10 through 17 reflect the right foot sensors, and columns 18 and 19 denote the total force beneath the left and right foot, respectively [25].

The dataset integrates recordings from three distinct subgroups based on prior clinical studies: Galit Yogev et al. (Ga), who focused on dual-task walking to explore cognitive-motor interaction; Hausdorff et al. (Ju), who investigated the impact of rhythmic auditory stimulation on gait patterns; and Frenkel-Toledo et al. (Si), who collected treadmill-based gait data under controlled conditions [11]. These subgroups contribute complementary insights into different gait characteristics relevant to PD diagnosis.

All participants were asked to walk for approximately two minutes at a self-selected pace while wearing the instrumented footwear. The dataset also includes clinical annotations such as disease status (PD or control) and Parkinson’s severity ratings based on the Hoehn and Yahr (HY) scale [11,26,27]. The HY scale characterizes PD progression across seven discrete stages. Stage 0 denotes the absence of clinical signs, while Stage 1 reflects very mild, unilateral involvement. Stage 1.5 introduces unilateral symptoms with axial signs, and Stage 2 denotes bilateral involvement without balance impairment. Stage 2.5 represents a mild bilateral condition revealed by the pull test. In Stage 3, postural instability emerges, although patients remain independently ambulatory. Stage 4 is marked by severe disability, rendering unaided standing or walking difficult, and Stage 5 denotes dependency on a wheelchair or assistance for mobility [4]. Based on the data in this study, Table 1 presents the number of participants classified according to the HY rating scale.

**Figure 3:**
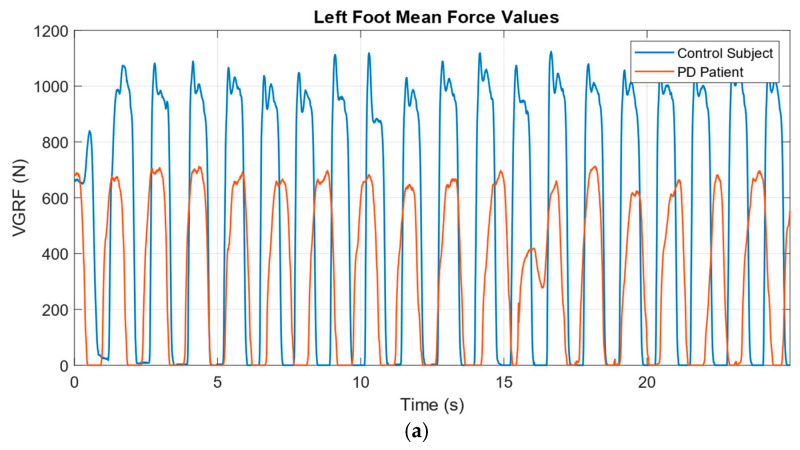

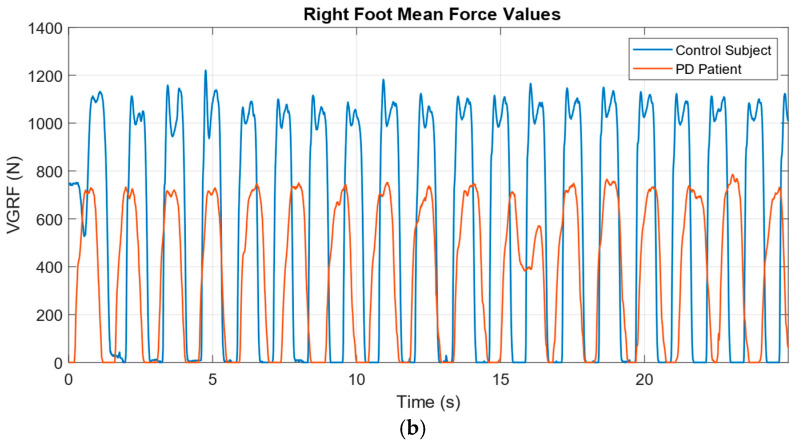
VGRF patterns obtained from the left (**a**) and right (**b**) feet of a control subject and a PD patient.

Mean VGRF waveforms from a control subject and a PD patient are depicted in Figure 3. Figure 3a shows the force trajectories under the left foot, while Figure 3b presents the corresponding right foot signals over a 25 s interval. As observed, the control subject exhibits more regular and higher-amplitude force profiles compared to the attenuated and irregular patterns of the PD subject.

### 2.2. Data Normalization and Windowing

As part of the data pre-processing phase, min-max normalization was applied to scale all numerical features to the [0, 1] range. This technique prevents features with larger numeric ranges from dominating the learning process and improves the stability and convergence of gradient-based optimization algorithms used in deep learning models [28]. The normalization was performed using the following standard formula:

(1) $$x^{\prime }=\;\frac{x-x_{min}}{x_{max}-x_{min}}$$

where x is the original value, $x_{min}$ and $x_{max}$ represent the minimum and maximum values of the signal, respectively, and $x^{\prime }$ denotes the normalized value. After normalization, the continuous signals were partitioned into fixed-length segments of 5, 10, 15, 20 and 25 s. Each segment (window) was treated for downstream feature extraction and classification, allowing the model to capture both short-term and longer-term temporal dynamics across multiple time scales. Similar durations have been used in previous studies [27] and in this work, they were selected both to examine the effect of different segment lengths on model performance and to ensure that each window includes sufficient gait activity, including multiple stride cycles, even in patients with irregular gait patterns.

### 2.3. Deep Learning

Deep learning is a subset of machine learning that employs multi-layered neural networks to automatically learn hierarchical representations from raw data. By leveraging large datasets and computational power, deep learning models can capture complex patterns and dependencies, making them particularly suitable for tasks such as image recognition, speech processing, and biomedical signal classification. In this study, deep learning is utilized to analyze gait signals for PD classification and severity assessment.

#### 2.3.1. Convolutional Neural Network (CNN)

Convolutional Neural Networks (CNNs) are a class of deep learning architectures that have shown remarkable success in tasks involving image analysis and time–frequency signal representation. CNNs operate by applying local filters to the input data, enabling the automatic detection of spatial and temporal patterns. Structurally, a CNN consists of sequentially arranged convolutional layers, nonlinear activation functions, pooling layers, and fully connected layers [29].

The convolutional layers apply learnable filters to local regions of the input, effectively capturing low-level features such as edges and textures. Mathematically, the two-dimensional convolution operation between an input matrix *X* and a kernel *W* is expressed:

(2) $$S\left(i,j\right)=\left(X*W\right)\left(i,j\right)=\sum_{m}\sum_{n}X(i+m,j+n)\cdot W(m,n)$$

This operation forms the foundation of feature extraction in CNNs. Following convolution, activation functions—most commonly the Rectified Linear Unit (ReLU)—introduce non-linearity into the model:

(3) $$f\left(x\right)=max(0,x)$$

Subsequent pooling layers reduce the dimensionality of feature maps, promoting translation invariance and improving computational efficiency. Finally, fully connected layers combine the extracted features and perform classification tasks.

**Figure 4:**
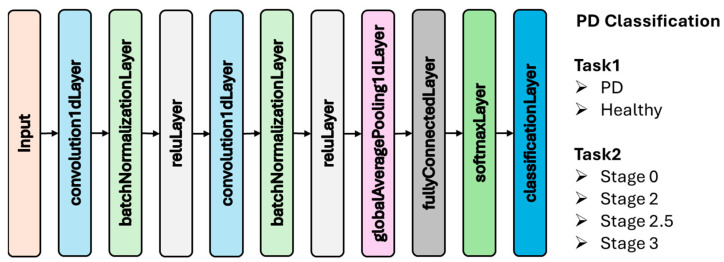
CNN architecture employed in this study.

In this study, the VGRF signals obtained from the gait data of Parkinson’s patients and healthy individuals were directly used as input to the CNN. Rather than transforming the signals into time–frequency representations, the network was trained end-to-end to learn discriminative features from the temporal patterns present in the original signals. This approach enables the model to capture fine-grained gait characteristics such as stride length variability, asymmetric force distribution, and inconsistent phase transitions relevant to PD classification and severity estimation. The CNN architecture used in this study is shown in Figure 4. This architecture was employed for two distinct tasks: (1) binary classification to distinguish between PD patients and healthy individuals, and (2) multi-class classification to estimate disease severity based on HY staging (Stage 0, Stage 2, Stage 2.5, and Stage 3). These two tasks were analyzed separately using the same CNN backbone, enabling focused evaluation of both diagnostic and staging performance.

CNNs offer a powerful solution for modeling spatially and temporally structured biomedical data. Their capacity to learn from raw signals without requiring handcrafted features makes them particularly suited for real-time and non-invasive diagnostic applications. Previous studies have demonstrated the efficacy of CNN-based models in analyzing gait signals for the detection and staging of neurodegenerative disorders [30].

#### 2.3.2. Long Short-Term Memory (LSTM)

Long Short-Term Memory (LSTM) networks are a specialized form of recurrent neural networks (RNNs) designed to model sequential data with long-range dependencies. Traditional RNNs often suffer from vanishing or exploding gradients when processing long sequences, making them inadequate for capturing temporal dynamics in many real-world signals. LSTM networks overcome this limitation by introducing a memory cell and gating mechanisms that regulate the flow of information across time steps.

An LSTM cell consists of four main gates (forget, input, memory cell update and output) that control the update and preservation of the cell state [31]. The mathematical operations involved in an LSTM unit are as follows:

Forget gate:

(4) $$f_{t}=\sigma (W_{f}\cdot \left[h_{t-1},x_{t}\right]+b_{f})$$

Input gate and candidate cell state:

(5) $$i_{t}=\sigma (W_{i}\cdot \left[h_{t-1},x_{t}\right]+b_{i})$$

(6) $${\tilde{C}}_{t}=tanh\;(W_{C}\cdot \left[h_{t-1},x_{t}\right]+b_{C})$$

Memory cell update:

(7) $$C_{t}=f_{t}\cdot C_{t-1}+i_{t}\cdot {\tilde{C}}_{t}$$

Output gate and hidden state:

(8) $$o_{t}=\sigma (W_{o}\cdot \left[h_{t-1},x_{t}\right]+b_{o})$$

(9) $$h_{t}=o_{t}\cdot tanh\;(C_{t})$$

Here, $x_{t}\;$ denotes the input at time step *t*, $h_{t-1}\;$ is the hidden state from the previous time step, and $C_{t}$ represents the memory cell. The sigmoid function σ(·) constrains the outputs between 0 and 1, enabling the network to selectively retain or discard information.

**Figure 5:**
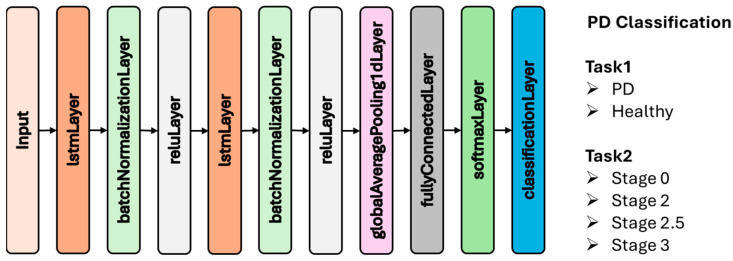
LSTM architecture employed in this study.

In the context of this study, the VGRF signals were used directly as input sequences to the LSTM network. Unlike models that rely on pre-extracted statistical or frequency-domain features, LSTM networks can learn temporal dependencies inherent in the raw gait signals, such as step intervals, swing phase durations, and bilateral asymmetries, which are critical for identifying motor impairments associated with PD. Figure 5 illustrates the LSTM architecture applied in this study. This network structure was utilized for two independent classification tasks: distinguishing between Parkinson’s patients and healthy controls, and determining disease severity based on HY stages. Each task was trained and evaluated separately to ensure targeted analysis of both diagnostic accuracy and stage-level classification performance.

This modeling approach is particularly suitable for detecting subtle, time-dependent gait variations that may not be evident in static features. By capturing the progression of biomechanical changes across gait cycles, the LSTM architecture provides a powerful tool for both PD classification and severity estimation based on temporal characteristics. Recent studies have shown that LSTM networks can outperform traditional classifiers in gait-based PD analysis, owing to their capacity to model complex, time-varying dependencies in sensor-derived data [32].

### 2.4. Bayesian Optimization

In ML and DL, the performance of a model is highly sensitive to its hyperparameters—those settings that govern the structure or learning behavior of the algorithm but are not learned directly from data. Efficient hyperparameter tuning is therefore essential to maximize model generalization. In this study, we employed Bayesian Optimization as a principled, sample-efficient method for selecting optimal hyperparameter configurations. Bayesian Optimization is a global optimization strategy that is particularly well-suited for optimizing black-box, non-convex, and computationally expensive functions where gradient information is unavailable or unreliable [33]. It constructs a probabilistic surrogate model of the objective function, typically a Gaussian Process (GP), and uses this model to guide the exploration of the hyperparameter space.

The core idea behind Bayesian Optimization is to balance exploration (sampling from regions with high uncertainty) and exploitation (focusing on regions likely to yield improved performance) via an acquisition function. At each iteration, the next hyperparameter set to evaluate is chosen by maximizing the acquisition function, which quantifies the expected utility of sampling a given point. Common acquisition functions include Expected Improvement (EI), Upper Confidence Bound (UCB), and Probability of Improvement (PI) [33,34].

Formally, given an objective function f:X→R where X is the space of hyperparameters, the optimization process involves:

1. Building a posterior distribution over f using observations $D={\left\{\left(x_{i},f\left(x_{i}\right)\right)\right\}}_{i=1}^{n}$
2. Selecting the next point $x_{n+1}$ by maximizing the acquisition function:

(10) $$x_{n+1}=\arg\;\underset{x\in X}{\max}\;\alpha (x;D)$$

where α(·) denotes the acquisition function.

In this work, Bayesian Optimization was applied to tune key hyperparameters of the classification models, including network architecture parameters and optimization settings. The objective function was defined as the validation accuracy obtained via k-fold cross-validation, ensuring robust and unbiased performance estimation. The optimization process continued iteratively until convergence criteria were met or a predefined number of evaluations was reached. Compared to traditional methods such as grid search or random search, Bayesian Optimization significantly reduced the number of required function evaluations while consistently identifying high-performing configurations. Its use was particularly valuable given the high dimensionality and training cost of deep learning models considered in this study.

### 2.5. Performance Evaluation

To assess the effectiveness and generalizability of the proposed PD classification models, a comprehensive performance evaluation was conducted using standard classification metrics. These include Accuracy (ACC), Precision (P), Recall (also known as Sensitivity, S), F1-Score, and the Area Under the Receiver Operating Characteristic Curve (AUC). These metrics collectively provide insight into the model’s ability to differentiate between Parkinson’s patients and healthy controls across various gait signal representations. The evaluation was performed using 10-fold cross-validation, which helps minimize overfitting and provides a robust estimate of model performance. In each fold, the dataset was partitioned into training and testing subsets such that each instance was used exactly once for testing. Mean values and standard deviations across folds were reported to ensure statistical stability [35,36,37]. The performance metrics are defined as follows:

(11) $$ACC=\frac{TP+TN}{TP+TN+FP+FN}\times 100$$

where *TP* is true positives, *TN* is true negatives, *FP* is false positives, and *FN* is false negatives.

(12) $$P=\frac{TP}{TP+FP}\times 100$$

(13) $$S=\frac{TP}{TP+FN}\times 100$$

(14) $$F1-score=2\times \frac{Precision\times Recall}{Precision+Recall}\times 100$$

AUC summarizes the model’s ability to distinguish between classes at various threshold settings. A value closer to 1 indicates superior discrimination capability. All computations were carried out in MATLAB R2024b. Models were trained using gait signal segments derived from VGRF data. Classification performance was evaluated based on statistical features extracted from decomposition-based representations of the gait signals.

## 3. Results

In this study, VGRF signals collected from both feet during walking were directly fed into CNN and LSTM models to both detect PD and estimate its severity. All experiments were carried out in MATLAB R2024b on a workstation equipped with an HP laptop (HP Inc., Palo Alto, CA, USA) with Intel Core i7-13700H CPU, 32 GB of RAM, and an 8 GB NVIDIA RTX 4070 GPU.

Table 2 presents the optimal hyperparameter configurations obtained for LSTM and CNN models across different time window lengths (5, 10, 15, 20, and 25 s) for both binary (PD vs. Control) and multi-class (severity level) classification tasks. In this study, Bayesian optimization was employed to determine the best-performing hyperparameters for each time window and model type. Compared to traditional grid search, Bayesian optimization offers a more efficient and cost-effective solution by constructing a surrogate model of the objective function and selecting promising hyperparameter sets based on acquisition functions. For each time window, the optimization process was constrained to a maximum of 30 objective evaluations and was carried out using 5-fold cross-validation, where the goal was to minimize the average validation error, defined as (1—accuracy). For LSTM models, the hyperparameters subjected to optimization included the initial learning rate [10^−4^, 10^−2^], the number of hidden units [32, 128], and the mini-batch size [16, 128]. In the case of CNNs, the search space covered the initial learning rate [10^−4^, 10^−2^], the number of filters in the convolutional layers [16, 128], filter size [3, 11], and the mini-batch size [16, 128]. The optimization was carried out using the *bayesopt* function included in MATLAB’s Statistics and Machine Learning Toolbox, with the expected-improvement-plus acquisition function. All DL models were implemented using MATLAB R2024b’s Deep Learning Toolbox.

Model training and evaluation were conducted via five-fold cross-validation: for each fold, VGRF windows of 5, 10, 15, 20, and 25 s were partitioned into training and test subsets, stratified by both PD diagnosis and HY stage. In MATLAB R2024b, both CNN and LSTM networks were trained according to the hyperparameter settings listed in Table 2. At each epoch, the networks were optimized first for the binary “healthy versus PD” task and then for the four-way PD staging task. Final performance metrics were obtained on the held-out test split of each fold, and results were averaged across all five folds for each time-window configuration. Bayesian optimization played a crucial role in enhancing model performance by systematically identifying optimal hyperparameter configurations. Across all analyses, the optimized models achieved approximately 1–2% higher accuracy compared to their non-optimized counterparts, along with consistent improvements in other performance metrics, demonstrating superior generalization capability.

Table 3 shows that window length has a stronger impact on PD gait classification performance than the choice of model. Using a 10 s sliding window, the LSTM achieves near-perfect results with the highest accuracy and AUC values, while the CNN also reaches its peak performance at this window length but remains consistently less effective across all configurations. Even short 5 s segments provide sufficient temporal information for accurate classification, particularly for the LSTM, whereas the CNN shows a more noticeable decline. As the window length increases beyond 15 s, both models experience a gradual drop in accuracy, F1-score, and AUC, likely due to the dilution of disease-specific gait characteristics. These results indicate that a 10 s window combined with an LSTM offers an optimal balance between data efficiency and classification performance, while longer windows may reduce the model’s ability to capture relevant gait dynamics.

Table 4 presents the results of PD severity classification based on multi-class labeling, where each instance is assigned to a specific stage of the disease. Similar to the binary classification task, the LSTM consistently outperforms the CNN across all window lengths in terms of accuracy, precision, recall, F1-score, and AUC. The highest performance is observed at the 5-s window, where the LSTM reaches an accuracy of 98.24% and an AUC of 0.999, while the CNN achieves 96.62% accuracy and 0.998 AUC, indicating that this window length provides the most effective temporal resolution for distinguishing between different severity levels.

At the 5-s window, both models maintain top-tier classification performance, suggesting that even brief gait segments contain rich discriminative information regarding disease severity. However, as the window length increases beyond 15 s, there is a consistent decline in all metrics for both models, particularly for the CNN. The drop is most pronounced at 25 s, where the CNN shows the weakest performance across all metrics (85.39% accuracy, 0.975 AUC), implying a loss of relevant temporal features over extended periods. These findings indicate that, for severity classification tasks, shorter windows (especially 5 s) are the most effective, and recurrent architectures such as LSTM are better suited to capturing the nuanced progression-related patterns in gait dynamics.

To further validate the comparative results, statistical significance testing was conducted using paired t-tests across all experimental configurations. The analyses confirmed that the performance differences between the LSTM and CNN models were statistically significant (*p* < 0.01) in both PD binary classification and HY stage classification tasks.

**Figure 6:**
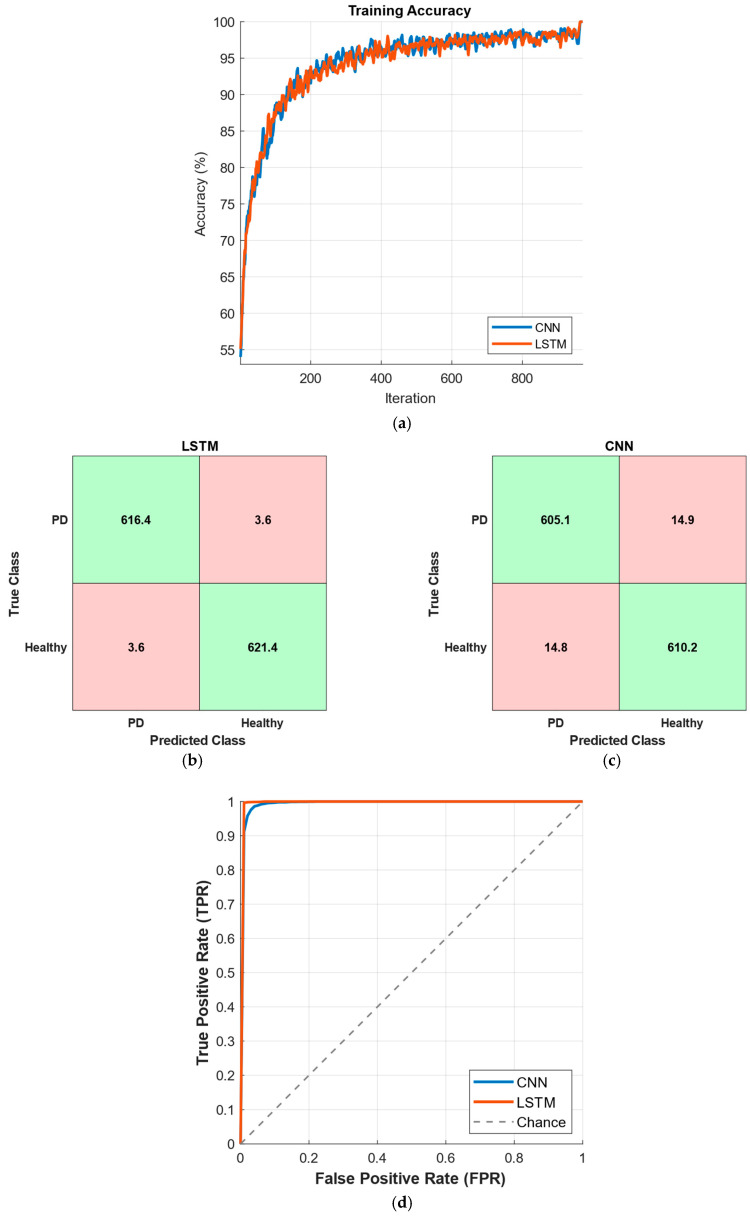
Performance of CNN and LSTM models for PD prediction for a time window of 10 s, with (**a**) training accuracy values, (**b**) confusion matrix for LSTM, (**c**) confusion matrix for CNN, and (**d**) ROC curves.

In Figure 6, the results corresponding to the best-performing time window of 10 s are presented. As shown in Figure 6a, both CNN and LSTM models exhibit a steady increase in training accuracy, converging above 95% after approximately 200 iterations. The average confusion matrices in Figure 6b,c indicate that both models achieve high true positive and true negative rates, with misclassification rates remaining below 4% for LSTM and slightly higher for CNN. The ROC curves in Figure 6d confirm the strong discriminative power of both models, with areas under the curve (AUC) approaching unity, highlighting their effectiveness in distinguishing Parkinson’s disease patients from healthy individuals.

**Figure 7:**
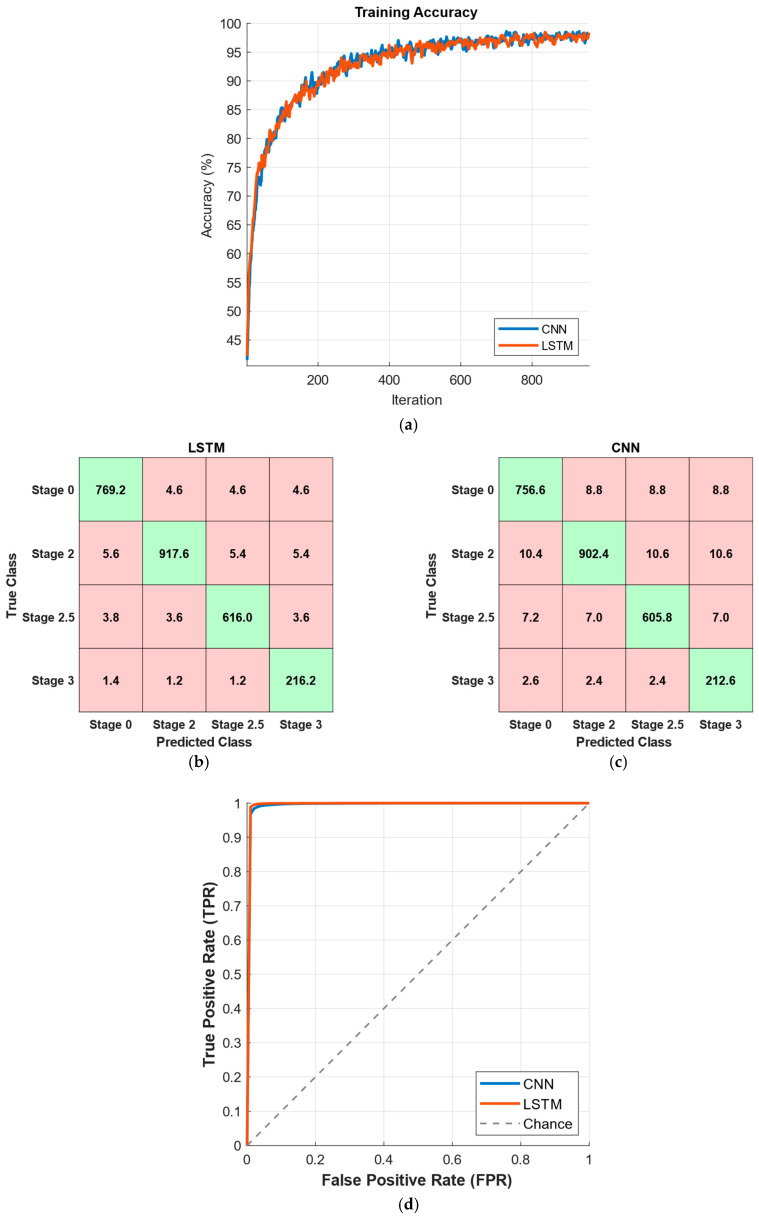
Performance of CNN and LSTM models for HY stage prediction for a time window of 5 s, with (**a**) training accuracy values, (**b**) confusion matrix for LSTM, (**c**) confusion matrix for CNN, and (**d**) ROC curves.

In Figure 7, the results corresponding to the best-performing time window of 5 s for predicting Hoehn and Yahr (HY) stages in Parkinson’s disease are presented. Figure 7a shows the training accuracy curves for CNN and LSTM models, both exhibiting a consistent increase in accuracy and converging close to 100% after approximately 300 iterations. The average confusion matrices in Figure 7b,c demonstrate that both models effectively classify the four HY stages, with high true positive rates across most classes and slightly lower performance in distinguishing adjacent stages. The ROC curves in Figure 7d indicate excellent discrimination capability for both models, with areas under the curve (AUC) values approaching 1.0, confirming their robustness in multi-class stage prediction.

**Figure 8:**
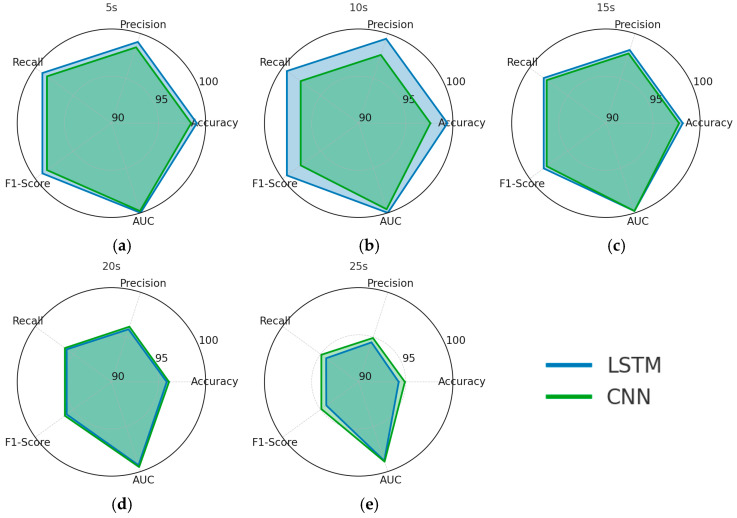
PD binary classification performance by time windows, with (**a**) 5 s, (**b**) 10 s, (**c**) 15 s, (**d**) 20 s, and (**e**) 25 s.

As illustrated in Figure 8, both LSTM and CNN achieve high AUC values at shorter time windows (5 s and 10 s), indicating strong discriminative capability in distinguishing PD patients from healthy controls when sufficient temporal resolution is preserved. However, as the window length increases beyond 15 s, a more pronounced decline is observed in the CNN’s performance across all evaluation metrics—particularly accuracy, recall, and F1-score—compared to the LSTM. This sharper degradation suggests that CNN is less effective at capturing long-range temporal dependencies in gait dynamics, whereas the LSTM maintains higher performance consistency.

**Figure 9:**
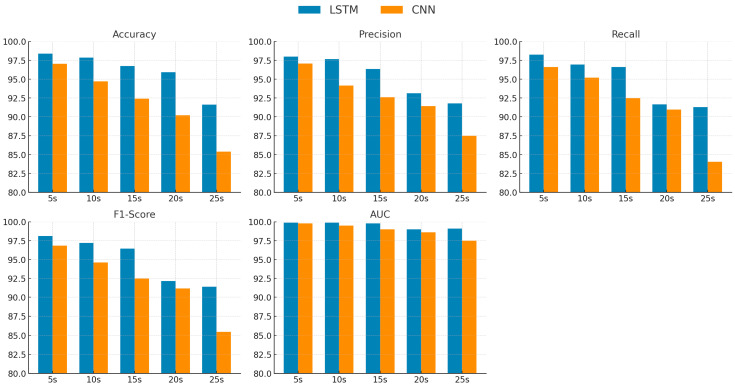
PD severity classification performance by time windows.

As shown in Figure 9, the grouped bar charts present a detailed comparison of LSTM and CNN performance in PD severity classification across five different time-window lengths (5 s, 10 s, 15 s, 20 s, and 25 s). For all evaluation metrics—accuracy, precision, recall, F1-score, and AUC—the LSTM consistently outperforms the CNN, with the performance gap becoming more pronounced as the window length increases beyond 15 s. This trend indicates that LSTM is more effective in modeling the complex temporal dependencies required for multi-class severity prediction, particularly when longer gait segments are analyzed. In contrast, the CNN exhibits a sharper decline in performance at extended windows, suggesting a reduced ability to capture progression-related temporal patterns over prolonged time frames.

## 4. Discussion

PD is a progressive neurodegenerative disorder that primarily affects motor function, with symptoms such as tremor, rigidity, bradykinesia, and postural instability. Accurate and early identification of PD is crucial, as it enables timely therapeutic interventions that can significantly slow disease progression and improve quality of life [38]. Diagnosis of PD typically relies on clinical examination, supported by tools such as the UPDRS and HY staging. However, these methods are often subjective and can vary depending on clinician expertise [3]. In recent years, gait analysis has emerged as a promising, objective biomarker for PD detection and severity assessment. Gait disturbances, including reduced stride length, increased variability, and asymmetry, are among the earliest observable symptoms of PD and often precede a formal diagnosis [39]. Numerous studies have shown that temporal and spatial features derived from gait signals contain discriminative patterns that can be leveraged for both binary classification and multi-class staging of the disease [40,41,42]. In addition to these technical advances, the clinical translation of gait-based deep learning models offers considerable potential. Wearable sensor systems have been increasingly recognized for their role in early diagnosis, body motion analysis, and long-term monitoring in PD. Their integration into telemedicine and home-based monitoring platforms further enables remote and continuous assessment, which may complement expert neurologist evaluation and extend care access to underserved populations. Moreover, the use of wearable sensors in clinical practice provides a more objective and ongoing evaluation of motor symptoms during real-world daily activities, reducing reliance on subjective ratings or episodic clinical assessments [43,44,45]. Future work will involve clinical validation with independent real-world cohorts to further assess the applicability and robustness of the proposed framework in routine practice.

In this study, we propose a deep learning-based framework that utilizes gait signals as input for PD classification. The continuous gait data were segmented into overlapping time windows of varying lengths (5, 10, 15, 20, and 25 s), and these segments were fed into LSTM and CNN models. To optimize model performance, Bayesian optimization was employed to tune hyperparameters for each configuration. Our experimental results demonstrate that the LSTM model achieved a peak binary classification accuracy of 99.42%, with corresponding precision, recall, and F1-score all exceeding 99%, and an AUC of 1.000 at the 10-s window. For the multi-class severity classification task, the best LSTM configuration attained 98.24% accuracy, 97.47% precision, 98.25% recall, and an AUC of 0.999 at the 5-s window. CNN models also showed competitive performance, particularly in shorter windows, with accuracies reaching up to 98.46% for binary classification and 96.62% for severity classification. The results highlight the potential of deep learning architectures to learn discriminative patterns directly from unprocessed gait data, enabling precise detection and staging of PD.

A comprehensive comparison of our approach with previous studies is presented in Table 5, which summarizes key works in the literature involving PD classification based on gait features. These studies vary in terms of feature extraction methods, model complexity, and input representations—ranging from handcrafted statistical features [11,46] to spatiotemporal transformations and deep CNN variants [47,48,49]. While many of these works report strong classification performance, they often rely on manually engineered features, domain-specific preprocessing steps, or specialized architectures such as ResNet [47] or DarkNet-CNN [48].

Compared to studies summarized in Table 5, the results obtained in our work appear to be promising. For instance, while ref. [50] reported 98.6% and 96.6% accuracy for binary and severity classification, respectively, our LSTM model—optimized via Bayesian search—achieved up to 99.42% and 98.24% in the corresponding tasks. Notably, ref. [11] achieved 99.1% accuracy using VMD-derived statistical features and ref. [51] reported 99.11% accuracy using 66 spatiotemporal features, whereas our optimized LSTM model reached 99.42% in binary classification and 98.39% in multi-class severity classification directly from raw gait signals. Although handcrafted feature extraction methods can deliver strong performance, our results demonstrate that deep learning can achieve comparable or superior accuracy while offering greater adaptability and reduced preprocessing requirements. The full classification outcomes across all time windows are provided in Table 3 and Table 4, where the LSTM and CNN models are evaluated under varying temporal resolutions. The results reveal not only the strong classification performance of the proposed models but also their robustness and adaptability to changes in input sequence length. These findings validate the effectiveness of optimization-driven deep learning models for gait-based PD diagnosis and progression assessment, while also highlighting their practical advantages in terms of simplicity and scalability compared to feature-intensive methods in the literature.

The study has several limitations. First, all results were obtained from a single publicly available dataset collected under specific recording conditions; although k-fold cross-validation mitigates overfitting within this cohort, it does not substitute for external validation on independent cohorts. Second, the severity distribution is skewed toward earlier stages, which can bias multi-class performance estimates. In addition, the dataset used for HY stage classification exhibits an imbalance, with only ten patients in stage 3 and the majority of subjects in earlier stages or controls. This distribution may influence multi-class performance estimates, particularly for underrepresented stages, and should be taken into account when interpreting the findings. Accordingly, the comparative metrics in this table should be interpreted with caution. Future work could validate the models on independent datasets collected under heterogeneous protocols and refine the training and evaluation procedures to better reflect clinical prevalence and decision needs.

**Table 5:** Summary of existing gait-based methods for PD diagnosis and severity assessment.

References	Feature Extraction Method	Machine/Deep Learning Model	Performance
[11]	VMD based 5 statistical features	CNN	The model achieved an accuracy of 99.1%, with sensitivity and specificity both reaching 100%.
[46]	7 statistical parameters belonging to the time and frequency domain	CNN	The models achieved AUC scores between 0.65 and 0.94, with sensitivity ranging from 52% to 92%, specificity from 51% to 95%, and precision between 30% and 62%.
[52]	Spatiotemporal gait features	Random Forest	The Random Forest model achieved 76% accuracy, 79% sensitivity, 70% specificity, %79 F1 score, and an AUC of 0.75.
[51]	Static and Dynamic Spatiotemporal Gait 66 Features	FNN	The FNN model attained an overall accuracy of 99.11%, along with a recall of 98.78%, a precision of 99.96%, and an F1-score of 99.23%.
[47]	Spatiotemporal gait features, polynomial transformation, 3D representation	PD-ResNet	The model achieved 95.51% accuracy overall, and 92.03% accuracy in classifying PD severity, with precision, recall, specificity, and F1-scores above 90% in both tasks.
[48]	Multiple Feature Evaluation Approach	Darknet CNN	The proposed DarkNet-CNN model achieved outstanding performance with 97.54% accuracy, 94.35% sensitivity, 89.67% specificity, 92.45% precision, and an F1-score of 91.45%.
[49]	Force-domain statistical features	CNN	The proposed method achieved up to 97.32% accuracy with XGBoost and 98.41% with deep learning.
[50]	Gait signals	LSTM	The Adam-optimized LSTM achieved 98.6% accuracy for binary classification and 96.6% for multi-class classification.
This Study	Gait signals	LSTM and CNN	The LSTM model, optimized via Bayesian optimization, achieved 99.42% accuracy for binary classification and 98.24% accuracy for multi-class classification of PD severity. CNN and LSTM architectures were evaluated across different time windows ranging from 5 to 25 s.

## 5. Conclusions

This study aimed to predict both the presence and the severity of PD by segmenting VGRF signals into fixed time windows of 5, 10, 15, 20 and 25 s and analyzing them with Bayesian optimized LSTM and CNN models. The LSTM model delivered its highest accuracy as 99.42% with AUC equal to 1.000 in binary classification at the 10 s window and reached 98.39% accuracy with area under the curve equal to 0.999 in HY staging at the 5 s window. The CNN model achieved 98.24% accuracy with AUC equal to 0.998 in binary discrimination and 97.05% accuracy with AUC equal to 0.998 in multiclass staging. Both approaches-maintained accuracy above 90% across all window sizes; to illustrate, at the 20 s window the LSTM and CNN models recorded 95.87% and 96.11% accuracy respectively.

These results highlight the promise of noninvasive VGRF based methods for Parkinson’s diagnosis and staging. Future work should explore hybrid architectures that combine CNN and LSTM frameworks to leverage their complementary strengths. In addition, integrating multiple gait parameters using multiple sensors and expanding the data set with recordings from varied daily life activities will improve model generalizability and robustness in real world scenarios.

## Figures and Tables

**Table 1:** Number of subjects in the PD database stratified by Hoehn–Yahr severity scale.

Stage 0	Stage 2	Stage 2.5	Stage 3
73	56	27	10

**Table 2:** Hyperparameter configurations of CNN and LSTM models for different time windows and classification tasks.

Task	Window (s)	Model	Initial Learn Rate	Mini Batch Size	Num Filters	Filter Size	Hidden Size
PD Binary Classification	5	LSTM	0.0010	32	-	-	64
		CNN	0.0010	32	64	5	-
	10	LSTM	0.0008	48	-	-	96
		CNN	0.0009	48	96	7	-
	15	LSTM	0.0012	32	-	-	128
		CNN	0.0012	32	128	9	-
	20	LSTM	0.0015	64	-	-	100
		CNN	0.0014	48	128	11	-
	25	LSTM	0.0011	48	-	-	128
		CNN	0.0010	64	96	7	-
PD Multi Class Classification	5	LSTM	0.0013	32	-	-	80
		CNN	0.0012	32	64	5	-
	10	LSTM	0.0010	48	-	-	100
		CNN	0.0009	64	48	7	-
	15	LSTM	0.0017	64	-	-	64
		CNN	0.0015	32	32	9	-
	20	LSTM	0.0022	32	-	-	96
		CNN	0.0021	64	128	11	-
	25	LSTM	0.0011	48	-	-	128
		CNN	0.0010	48	96	7	-

**Table 3:** PD classification results.

Time Window (s)	Classifier	Accuracy	Precision	Recall	F1-Score	AUC
5	LSTM	99.06% ± 0.24	99.07% ± 0.24	99.06% ± 0.24	99.06% ± 0.24	1.000 ± 0.000
	CNN	98.46% ± 0.54	98.47% ± 0.54	98.46% ± 0.54	98.46% ± 0.54	0.998 ± 0.001
10	LSTM	99.42% ± 0.29	99.42% ± 0.29	99.42% ± 0.29	99.42% ± 0.29	1.000 ± 0.000
	CNN	97.61% ± 1.16	97.62% ± 1.15	97.61% ± 1.16	97.61% ± 1.16	0.996 ± 0.003
15	LSTM	98.16% ± 0.64	98.17% ± 0.64	98.16% ± 0.64	98.16% ± 0.64	0.998 ± 0.001
	CNN	97.77% ± 0.66	97.78% ± 0.65	97.77% ± 0.66	97.77% ± 0.66	0.998 ± 0.001
20	LSTM	95.87% ± 2.90	95.87% ± 2.90	95.87% ± 2.90	95.87% ± 2.90	0.993 ± 0.008
	CNN	96.11% ± 1.27	96.16% ± 1.27	96.11% ± 1.27	96.11% ± 1.27	0.995 ± 0.003
25	LSTM	94.25% ± 1.22	94.41% ± 1.26	94.27% ± 1.20	94.25% ± 1.21	0.988 ± 0.010
	CNN	94.90% ± 2.19	94.90% ± 2.19	94.90% ± 2.19	94.90% ± 2.19	0.989 ± 0.007

**Table 4:** PD HY severity classification results.

Time Window (s)	Classifier	Accuracy	Precision	Recall	F1-Score	AUC
5	LSTM	98.24% ± 0.48	97.47% ± 0.54	98.25% ± 0.78	97.85% ± 0.53	0.999 ± 0.000
	CNN	96.62% ± 0.39	95.23% ± 0.44	96.63% ± 0.53	95.88% ± 0.37	0.998 ± 0.001
10	LSTM	97.85% ± 0.67	97.67% ± 0.29	96.93% ± 2.29	97.21% ± 1.32	0.999 ± 0.000
	CNN	94.70% ± 1.30	94.16% ± 1.74	95.19% ± 1.24	94.61% ± 1.45	0.995 ± 0.001
15	LSTM	96.75% ± 1.25	96.34% ± 0.94	96.60% ± 0.73	96.45% ± 0.84	0.998 ± 0.001
	CNN	92.40% ± 1.71	92.60% ± 1.35	92.49% ± 1.99	92.47% ± 1.50	0.990 ± 0.003
20	LSTM	92.90% ± 4.15	93.12% ± 4.04	91.65% ± 6.03	92.15% ± 5.30	0.990 ± 0.012
	CNN	90.20% ± 1.95	91.44% ± 1.58	90.98% ± 1.41	91.16% ± 1.49	0.986 ± 0.003
25	LSTM	91.61% ± 1.77	91.78% ± 2.02	91.29% ± 2.27	91.38% ± 1.58	0.991 ± 0.002
	CNN	85.39% ± 3.34	87.49% ± 3.47	84.04% ± 3.65	85.47% ± 3.57	0.975 ± 0.008

## Data Availability

This study employs the publicly accessible dataset titled Gait in Parkinson’s Disease, which is available online via PhysioNet (https://physionet.org/content/gaitpdb/1.0.0/) (accessed on 20 June 2025).

## Abbreviations

The following abbreviations are used in this manuscript:

AUC	Area Under the Receiver Operating Characteristic Curve
CNN	Convolutional Neural Network
DL	Deep Learning
EMD	Empirical Mode Decomposition
FFT	Fast Fourier Transform
HY	Hoehn and Yahr
LBP	Local Binary Pattern
LS-SVM	Least Squares Support Vector Machines
LSTM	Long Short-Term Memory
ML	Machine Learning
PCA	Principal Component Analysis
PD	Parkinson’s Disease
UPDRS	Unified Parkinson’s Disease Rating Scale
VGRF	Vertical Ground Reaction Force
